# Potential profiling of self-management skills in older co-morbid patients

**DOI:** 10.1186/s12877-024-05137-4

**Published:** 2024-06-25

**Authors:** Wu Lanxin, Zhang Yan, Tian Yutong, Meng Lixue, Liu Li, Zhao Ting

**Affiliations:** Zhengzhou University School of Nursing and Health, Zhengzhou, Henan Province China

**Keywords:** Older people, Co-morbid patients, Self-management, Latent profile analysis

## Abstract

**Background:**

Under the general trend of global aging, geriatric comorbidity is increasingly common, which may have some impact on the quality of life of the older people. Self-management can effectively improve patient compliance, subjective initiative, and improve patient quality of life. However, the present situation of self-management in different old people is different. Therefore, this study classifies older co-morbid patients through potential profiling analysis, understands the category characteristics of self-management level of older co-morbid patients, and discusses the influencing factors of self-management level of different categories of older co-morbid patients, which can provide reference for personalized intervention programs for different comorbidity characteristics of elderly people in the future.

**Method:**

Through a cross-sectional study, 616 cases of older co-morbid patients in three districts of Zhengzhou City, Henan Province, were selected as survey subjects by using the whole cluster sampling method. The General Information Questionnaire, Chronic Disease Self-Management Scale, Health Literacy Scale, Electronic Health Literacy Scale, Collaborative Social Support Scale, and Health Empowerment Scale were used to conduct the survey.

**Results:**

The result of LPA shows that the self-management characteristics of older co-morbid patients should be classified into 3 categories: good self-management (19.4%), medium self-management(27.9%), and low self-management (52.7%). The results of multivariate logistic regression analyses show that literacy, religiosity, health literacy, e-health literacy, appreciative social support, and health empowerment are influential factors for self-management among older co-morbid patients (*p* < 0.05).

**Conclusion:**

There is obvious heterogeneity in the self-management level of older co-morbid patients. It is recommended that healthcare professionals give targeted interventions for their weaknesses according to the self-management characteristics of different categories of patients in order to enhance the self-management level of this population and improve their quality of life.

## Introduction

According to the data of the Seventh National Population Census of the National Bureau of Statistics in 2020, the older people population aged 60 years and above in China was 260 million, accounting for 18.70% of the total population [1]. It is expected that by 2050, China's older people population aged 60 years and older will approach 500 million, accounting for more than one-third of the total population [2]. As life expectancy increases, so does the number of older people with chronic diseases. Approximately 15.3% to 91.3% of the global population suffers from comorbidity [3]. According to recent data from the British Medical Journal (BMJ), comorbidity are predominantly age-driven in high-income countries around the world. Therefore, the elderly co-patients need urgent attention.With demographic changes, the proportion of the population with two or more diseases is steadily increasing [4]. The Report on Nutrition and Chronic Diseases in China (2020) points out that the aging of China's population is deepening, and the incidence of various chronic diseases, mainly hypertension, diabetes mellitus, and chronic obstructive pulmonary disease (COPD), is increasing, especially comorbidity in the older people [5]. Comorbidities, such as frailty and increased medication, result in decreased functional status and quality of life." It puts higher demands on health management of comorbidity.

Active self-management can make people's behavior change to health-related behavior, and improve their quality of life by improving their lifestyle and ability to cope with diseases. Self-management refers to a series of health actions taken by patients to cope with the disease, reduce the occurrence of complications, and mitigate the negative impact of the disease on their social activities and psychological factors [6]. Self-management can effectively improve patients' compliance, subjective initiative and quality of life [7]. In China, self-management of older patients with chronic diseases is still at a low level [8]. Most older have limited knowledge of their diseases, and poor management may lead to more complications and medical costs, as well as have an impact on their quality of life [9]. Studies have shown that self-management of chronic diseases can improve the health and quality of life of older patients with chronic diseases and reduce the incidence of complications [10].

Current research on self-management of elderly patients with co-morbidities mostly focuses on their overall level and influencing factors. However, these studies have mostly adopted a traditional variable-centered approach (e.g., regression analysis) [11, 12], ignoring the multidimensional features of self-management, the heterogeneity of sample and limiting the ability to precision intervene. Different comorbid elderly people have different self-management problems, so it is difficult to guide them through a unified program, and there is a lack of targeted intervention programs for different groups of people. Therefore, we can consider the classification and analysis of the heterogeneity of the population's self-management ability, in order to clarify the characteristics and influencing factors of self-management in different groups, and to promote targeted intervention plans in the later stage. And Latent profile analysis is an individual-centered statistical analysis method, which explains the association between external continuous variables through potential category variables and realizes the local independence between exogenous variables [13]. Importantly, this method allows for the identification of populations most in need of intervention and identifies where there is a need for intervention in different domains. Bajenaru L conducted a quality of life questionnaire on older patients, and divided the population into three categories through potential category analysis, which revealed the heterogeneity in the older population, which was conducive to the development of targeted intervention programs to improve their quality of life according to the different needs of older patients in the later stage [14].

The aim of this study is to classify the self-management categories of older co-morbid patients through LPA and analyzes their influencing factors. We anticipate that the finding of this research may help to develop tailored interventions based upon identified patterns, particularly for those with low level of self-management, to enhance self-management ability in this population.

## Method

### Study design and participants

The multi-stage stratified cluster sampling method was used, with a total of nine main urban districts in Zhengzhou City, and three urban districts, Jinshui District, Zhongyuan District, and Hi-tech District, were selected according to the high, medium, and low levels of economic development. One community was randomly selected from each of the three main urban areas according to the random number table method. A total of three survey points were obtained from Nanyang Road, Linsanzhai and Feng Yang Street communities. People who met the inclusion criteria in the three communities were included in the study:① age ≥ 60 years;② meet the WHO diagnostic criteria for hypertension, coronary heart disease, cerebral infarction, diabetes mellitus, chronic obstructive pulmonary disease (COPD), osteoarthritis, etc.; the type of disease was determined with reference to the health management records of community residents of the community health service centre;③ people with normal reading, comprehension, and expression who are able to assess their physical abilities. Exclusion criteria:① people with severe mental disorders;② people who are participating in other clinical trials. After receiving information about the study the participants signed the written informed consent to participate in our study, the voluntary nature of the study and their data anonymity and confidentiality were strictly assured. This study was conducted with the approval from the Ethics Committee of Zhengzhou University, (ZZUIRB2023-069).

In this study, the presenting survey sample size was calculated using the formula:$n=\frac{{\mu }_{\alpha }^{2} \rho (1-\rho )}{{\delta }^{2}}$ *deff, where* ρis the presenting rate of 60% of older co-morbid patients [15], δ is the permissible error, generally used = 0.1 × ρ. Considering the 15% invalid sample, the required sample size was 590. 650 questionnaires were distributed. 616 were validly returned.

## Instruments

### General information questionnaire

A general information questionnaire was designed based on a literature review and consultation with experts, encompassing participants’ demographic information.Such as age, gender, ethnicity, place of residence, education level, marital status, religious beliefs, monthly income, occupation, residence, medical payment method, and disease duration.

### Chronic disease self-management scale

Chronic Disease Self-Management Scale [16] was used to measure the cognitive ability, psychological quality, lifestyle, and treatment adherence of the study subjects. The scale contains 4 primary indicators and 40 secondary indicators. All items adopt the Likert5 rating method(1 = no, 5 = always). Higher scores mean higher self-management skills. The Cronbach's alpha coefficient of the scale was 0.919, with good reliability.

### Health literacy scale

The Health Literacy Scale for Patients with Chronic Diseases was used to measure patients' health literacy levels [17]. The scale consists of 24 entries with 4 dimensions: ability to obtain information, ability to communicate and interact, willingness to improve health, and willingness to provide financial support. All items adopt the Likert5 rating method (1 = almost impossible, 5 = no difficulty et al.). A score of less than 96 indicates a lack of health literacy, while a score of more than 96 indicates adequate health literacy. Higher scores mean higher health literacy. The scale Cronbach'αwas 0.864, good reliability and validity.

### Electronic health literacy scale

EHealth Literacy in Chronic Disease Patients [18] was used to determine the patient's ability to detect the quality of information on the Internet. The eHEALS scale consists of 8 entries, including three dimensions: ability to apply online health information and services (entries 1–5), judgemental ability (entries 6 and 7), and decision-making ability (entry 8). Each entry was scored on a 5-point Likert scale, Higher scores representing better e-health literacy, and a score of more than 32 being qualified. The Cronbach's alpha of the scale was 0.950, with good reliability.

### Health empowerment scale

The Chronic Disease Health Empowerment Scale [19] was used to measure the level of health empowerment of chronic patients. The scale consists of 5 dimensions: belief in responsibility, obtaining support, increasing knowledge, participating in treatment, and reconstructing oneself, with a total of 26 items. Each entry was scored on a 5-point Likert scale (1 = strongly disagree, 5 = strongly agree). Higher scores indicating a higher awareness of health empowerment. The scale Cronbach'αwas 0.927, indicating that the scale has good reliability.

### Comprehending the social support scale

Comprehending the Social Support Scale [20] was used to measure individuals' perceived level of social support from different sources. There are 12 items, including three dimensions of family support (items 3, 4, 8, and 11), friend support (items 6, 7, 9, and 12), and other support (1, 2, 5, and 10). Each item was scored on a seven-point scale from 1 (strongly disagree) to 7 (strongly agree). Higher scores indicating a higher level of social support felt by the individual. The scale is generally categorised into three levels, low support status (12–36 points), intermediate support status (37–60 points) and high support status (61–84 points). The total Cronbach's alpha coefficient for the scale was 0.899, and the split-half reliability was 0.878.

### Data collection and quality control methods

On the basis of obtaining the consent of the relevant community management personnel, the researcher used the staged cluster sampling method to select research subjects who met the criteria for distributing the questionnaire. Following the principle of voluntariness, a unified instruction was used to explain how to fill in the questionnaire and the precautions to be taken, and after filling in the questionnaire, it was uniformly collected by the researcher, and for those who could not fill in the questionnaire, the researcher asked for the answer and then filled it in for them. After collecting the questionnaires, the information collected was carefully reviewed and checked, and statistical software was used for data entry and statistical analysis.

### Statistical methods

Self-Management Scale as the exogenous variables to carry out the LPA. Initially, only 1 profile was assumed, and then the number of profiles was gradually increased and the parameters of each model were analysed to select the optimal model against the fitting index. Potential profiles were analysed with 3 types of fit indicators [13, 21] (i) Model fit test. The model fit test indicators are Akaike information criterion (AIC), Bayesian information criterion (BIC) and adjusted BIC (aBIC), the smaller the value, the better the model fit. (ii) The classification index is entropy, which takes the value of 0 ~ 1, and the closer to 1, the higher the accuracy. (iii) Likelihood ratio test indicators include LoMendell-Rubin likelihood ratio (LMR), Bootstrap likelihood ratio test (BLRT), *p* < 0.05 indicates that the kth category model is better than the k-1st category model. superior to the k-1st category model. Statistical analyses were performed using SPSS 25.0 software.Measurement information was expressed as mean ± standard deviation or M (P25, P75). Count data were expressed as frequencies and percentages, and comparisons between groups were made using the chi-square test. The results of category analysis of LPA were used as the dependent variable, and general information with statistical significance was screened as the independent variable for between-group comparisons, and multivariate logistic regression was used to analyse the influencing factors of different self-management categories of older people co-morbidities, and the difference was considered to be statistically significant at *p* < 0.05.

## Results

### General information, self-management status of older comorbid patients

A total of 616 older co-morbid patients are included in this study, 211 male and 405 female.78 people aged 60–69,279 people aged 70–79,259 people aged more than 80. 266 people have an education level of primary school or below. 193 people have an education level of junior high school 0.79 people have an education level of senior high school. 78 people have an education level of Specialist and above. The older co-morbid patients' self-management score was (128.66 ± 10.789), health literacy score (96.02 ± 15.063), e-health literacy score (30.87 ± 9.615), comprehension of social support score (88.14 ± 9.615), and health empowerment score (88.14 ± 7.893), as shown in Table 1.

**Table 1:** Self-management and other scale scores for older co-morbid patients

Variable	Score range	Score	Entry parity
**Self-management**	40–200	128.66 ± 10.789	3.22 ± 0.226
Cognitive ability	13–65	32.57 ± 6.779	2.51 ± 0.521
Psychological diathesis	6–30	20.83 ± 2.663	3.47 ± 0.444
Life style	14–70	48.97 ± 4.566	3.50 ± 0.336
Adherence to treatment	7–35	26.30 ± 2.353	3.76 ± 0.336
**Health literacy**	24–120	96.02 ± 15.063	4.00 ± 0.628
**E-health literacy**	8–40	20.87 ± 9.615	2.61 ± 1.202
**social support**	12–84	71.61 ± 7.741	5.97 ± 0.645
**Health empowerment**	26–130	88.14 ± 7.893	3.39 ± 0.304

### Results of potential profiling of self-management in older co-morbid patients

**Fig. 1:**
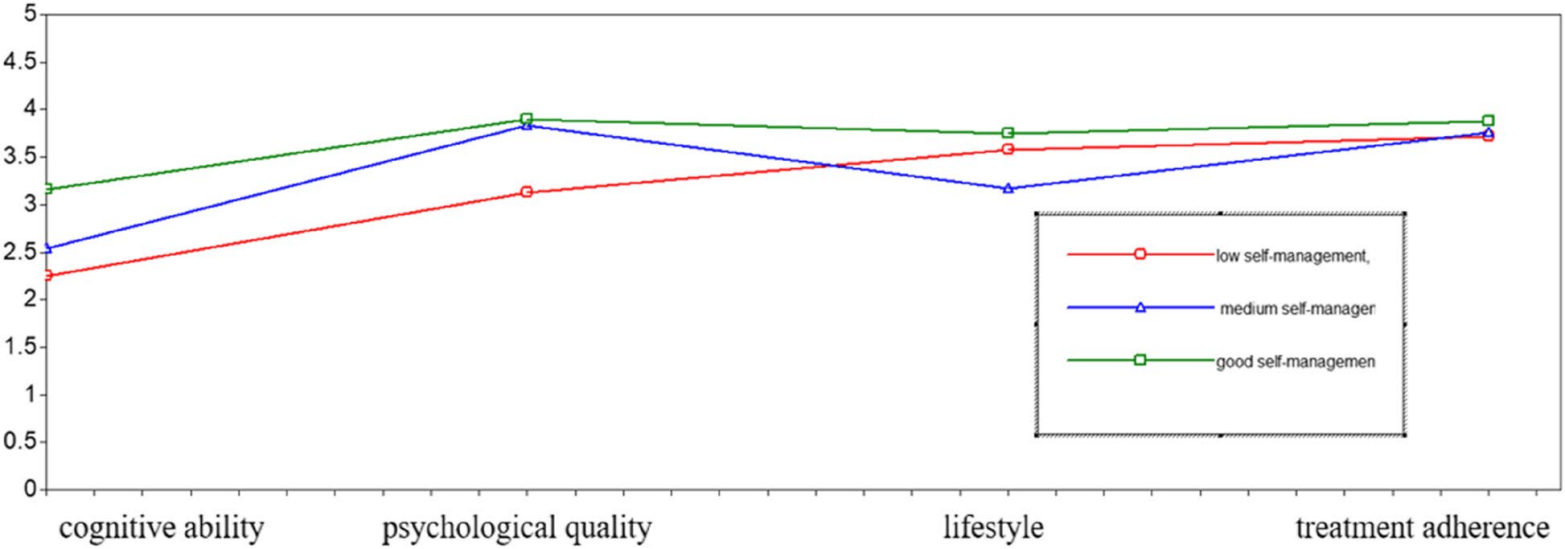
Distribution of 3 potential profile characteristics for self-management in older co-morbid patients

A total of 5 models were fitted to the profile analysis with the scores of the 4 dimensions of chronic disease self-management, and the fitting indexes of each model are shown in Table 2. With an increase in the number of latent profiles, the AIC, BIC, and ABIC gradually decreased, the entropy value increased gradually, in which the model 3 category was the largest. Therefore, the model of the 3 categories was selected as the best potential profile model for the self-management of older patients with co-morbidity. On the basis of the potential category model determination, the potential profiles of the 3 categories on the 4 dimensions of self-management were obtained (Fig. 1). A total of 118 cases (19.4%) were in category 1, which was named as good self-management type because the overall self-management level of patients in this category was high and the scores of all dimensions were higher than those of other categories. Category 2 consisted of 166 cases (27.9%), in which the overall self-management level of the patients was medium, and the scores of the lifestyle dimensions were significantly lower than those of other categories, so it was named the medium self-management type. Category 3 consisted of 322 cases (52.7%), the overall self-management level of patients in this category was low, and the scores of all dimensions were low, with cognitive ability being the lowest, so it was named self-management low type.

**Table 2:** Indicators of fit for a potential profile model of self-management in older co-morbid patients

Model	AIC	BIC	aBIC	Entropy	LMRT	BLRT	Categorical probability
1	2474.721	2510.107	2484.709	-	-	-	-
2	2208.67	2266.172	2224.899	0.828	0.0000	0.0000	0.532/0.468
3	2067.017	2146.635	2089.489	0.838	0.0000	0.0000	0.528/0.281/0.191
4	1958.839	2060.574	1987.553	0.835	0.0225	0.0000	0.272/0.294/0.255/0.178
5	1868.501	1992.352	1903.457	0.836	0.0007	0.0000	0.150/0.268/0.307/0.009/0.185

### Univariate analysis of potential self-management profiles of older comorbid patients

The differences between the three potential categories of self-management levels of older comorbid patients were statistically significant (*p* < 0.05) in terms of gender, education, religion, monthly income, pre-retirement occupation, and healthcare payment method, as shown in Table 3.

**Table 3:** Univariate analyses of general information and potential profiles of self-management in older co-morbid patients

Variable	Sum	C1	C2	C3	χ ^2^/F	*p*
**Gender**					8.977	0.011
Male	211	53 (25.1)	59 (28.0)	99 (46.9)		
Female	405	65 (16.1)	107 (26.4)	233 (57.5)		
**Age**					2.337	0.674
60–69	78	12 (15.4)	19 (24.4)	47 (60.2)		
70–79	279	75 (26.9)	59 (21.1)	145 (52.0)		
≥ 80	259	47 (18.1)	72 (27.8)	140 (54.1)		
**Nation**					4.519	0.104
Han	607	116 (19.1)	161 (26.5)	330 (54.4)		
Other	9	2 (22.2)	5 (55.6)	2 (22.2)		
**Current address**					5.466	0.065
City	494	101 (20.4)	138 (27.9)	255 (51.7)		
Village	122	17 (13.9)	28 (23.0)	77 (63.1)		
**Degree of education**					24.343	0.000
Primary school below	266	37 (13.9)	71 (26.7)	158 (59.4)		
Junior high school	193	39 (20.2)	41 (21.3)	113 (58.5)		
Senior high school	79	19 (24.1)	26 (32.9)	34 (43.0)		
Specialist and above	78	23 (29.5)	28 (35.9)	27 (34.6)		
**Marital status**					3.092	0.213
Married	458	95 (20.7)	123 (26.9)	240 (52.4)		
Unmarried	158	23 (14.6)	43 (27.2)	92 (58.2)		
**Religion**					10.297	0.006
Yes	31	3 (9.7)	16 (51.6)	12 (38.7)		
No	585	115 (40.4)	150 (25.6)	320 (54.0)		
**Monthly income**					17.662	0.001
< 1000	197	25 (12.7)	44 (22.3)	128 (65.0)		
1000–3000	42	13 (31.0)	10 (23.8)	19 (45.2)		
> 3000	377	80 (21.2)	112 (29.7)	185 (49.1)		
**Pre-retirement** **occupation**					16.145	0.003
Enterprises	409	92 (22.5)	119 (29.1)	198 (48.4)		
Public institutions peasant	172	21 (12.2)	40 (23.3)	111 (64.5)		
Liberal professions	35	5 (14.3)	7 (20.0)	23 (65.7)		
**Living situation**					11.518	0.074
Alone	87	20 (23.0)	27 (31.0)	40 (46.0)		
Live with spouse	292	58 (19.9)	85 (51.2)	149 (51.0)		
Live with children	163	22 (13.2)	37 (22.7)	104 (63.8)		
Live with spouse and children	74	18 (24. 3)	17 (23.0)	39 (52.7)		
**Medical** **Payment method**					16.145	0.003
Self-funded	35	5 (14.3)	7 (20.0)	23 (65.7)		
Urban health care	409	92 (22.5)	119 (29.1)	198 (48.4)		
Rural health care	172	21 (12.2)	40 (23.3)	111 (12.2)		
**Disease duration**					3.658	0.454
< 10	193	44 (22.8)	48 (24.9)	101 (52.3)		
10–20	310	53 (17.1)	83 (26.8)	174 (56.1)		
> 20	113	21 (18.6)	35 (31.0)	57 (50.4)		

*C1* Good self-management type, *C2* Medium self-management type, *C3* Low self-management type

### Multifactorial analysis of potential categories of older co-morbid patients

Multiple Logistic regression analyses were performed using the low self-management type group as the reference group. The results of the multiple Logistic regression are shown: Firstly, Elderly co-morbid patients with junior high school education are more likely to be categorised as low self-management types. Secondly, elderly co-morbid patients with religious affiliation are more likely to be categorised as having medium self-management type. Then, Health literacy, social support and health empowerment promote the self-management level of older co-morbid patients. As shown in Table 4.

**Table 4:** Analysis of factors influencing potential self-management profiles of older co-morbid patients

Variable	β	SE	Wald χ^2^	P	OR	95%CI
C1						
Intercept	-31.587	2.930	116.197	0.000		
Health literacy	0.041	0.014	8.854	0.003	1.042	1.014–1.071
Social support	0.080	0.022	13.717	0.000	1.083	1.038–1.130
Health empowerment	0.222	0.324	0.211	0.646	1.160	0.615–2.188
C2						
Intercept	-10.091	1.969	26.275	0.000		
Health literacy	0.031	0.09	11.047	0.001	1.032	1.013–1.051
E-health literacy	0.034	0.012	7.916	0.005	1.034	1.010–1.059
Social support	0.039	0.016	5.880	0.015	1.040	1.008–1.074
Health empowerment	0.036	0.017	4.597	0.032	1.037	1.003–1.072
Degree of education						
Junior high school	-0.799	0.354	5.101	0.024	0.450	0.225–0.900
Religion						
Yes	0.866	0.430	4.055	0.044	2.378	1.023–5.528

C1 represents the group with good self-management, C2 represents the group with medium self-management, and C3 represents the group with low self-management, with C3 as the reference group

## Discussion

The result of this study shows that the self-management average score of older comorbid patients is (3.22 ± 0.226). It is at an intermediate level but lower than the Qian Yan [22] etal findings on self-management ability of older diabetic patients (3.85 ± 1.38) points. The possible reasons for this are: with the increase in the number of diseases, the complexity of diagnosis and treatment protocols, the difficulty of disease management increases, and self-management ability decreases [23].The four dimensions of self-management: cognitive ability (2.51 ± 0.52), psychological quality (3.47 ± 0.44), lifestyle (3.50 ± 0.33), and treatment adherence (3.76 ± 0.34), shows that the scores of cognitive ability are relatively low. It is necessary to carry out education and training for the cognitive ability of the older to raise the importance of self-management of the older, and then improve the self-management ability of older patients with comorbidity.

In this study, three characteristic categories of self-management in older comorbid patients are identified based on LPA, and each fitting indicator indicates a good model fit, suggesting that there is significant variability in the level of self-management among older comorbid patients. Category 1 was good self-management (19.4%), which had higher scores on all dimensions than the other categories and has the smallest number of people. It is possible that this category has higher literacy, higher monthly income, and less material and mental stress. Therefore they will pay more attention to disease management-related content. It promotes patients' adoption of health-promoting behaviors, which is consistent with the results of the Shields study [24]. Category 2 is the medium level of self-management type (27.9%). The overall self-management is at a medium level, but the lifestyle score is lower than the other 2 categories. It may be that most of the older people in this group are over 70 years old and have a certain sense of self-management, but their ability to acquire scientific health knowledge is limited. They mostly manage with their own experience.It lacks a certain degree of scientificity, thus showing a medium self-management type. Category 3 is the low self-management type, accounting for the largest proportion of 52.7%. It may be due to the fact that most of the people in this category have a low level of education. They lack a proactive awareness of health care, have a more passive way of acquiring health knowledge, and have a limited ability to acquire health management. It leads to a low level of self -management [25]. Therefore, medical professionals need to actively communicate with patients, understand their self-management categories, strengthen the education of self-management weaknesses of different categories of people. For the elderly with low self-management, health education should be carried out in an easy-to-understand and acceptable way. For the elderly with medium self-management, help them establish a correct lifestyle and correct their bad health behaviors. At the same time, invite the elderly with good self-management to share their experiences with other categories of people, so as to improve the enthusiasm of other groups. By providing tailor-made education and training for different categories of people, it is conducive to the improvement of their self-management ability.

The result of this study shows that patients with a junior high school education level had a higher likelihood of belonging to the low self-management group (*p* < 0.05). It is Consistent with related research [26]. The reasons for this are analysed:This group of people had a certain level of knowledge, and their pre-retirement occupations were mostly workers, with a high level of daily work intensity.Therefore, they don’t pay enough attention to disease management, and ignored the daily management of their illnesses. It led to the overall low level of self-management. For this group of people, self-management publicity should be increased to enhance the importance of disease management. It is suggested that medical staff should consider the acceptance and compliance of patients with education level. For patients with low education level, peer education and exemplary role can be fully utilized to enhance patients' self-management awareness, and easy-to-understand pictures or audio-visual materials can be used for planned implementation and supervision [27]. At the same time, disease knowledge acquisition channels should be expanded and health education forms should be enriched to attract the interest of the older people and enhance the effect of health education, thus improving the self-management level of older people patients with comorbidity.

The results of this study shows that the older co-morbid patients with religious beliefs has a higher possibility of belonging to the group of medium self-management (*p* < 0.05). The possible reason is that elderly people with religious beliefs have a sense of faith. It helps to overcome the fear of disease, so as to better manage the disease. In addition, this group of people usually have peers with similar interests and a higher degree of social participation.It is conducive to the improvement of their self-management level through emotional comfort and experience sharing among peers. However, knowledge of health education is the key to the adoption of health behaviors by the older people [28].Therefore,the self-management level of older co-morbid patients with religious beliefs is at a medium level, while their lifestyle is still at a low level, It is necessary to be further improved to enhance the health knowledge ability of the older people, and then improve their self-management level. This suggests that healthcare professionals and patients' families can encourage the older people to participate in more social activities, cultivate their own hobbies and interests, and actively integrate into peer groups to help the older people alleviate mental stress, share their experiences of disease management and overcome their illnesses [11].

The results of this study shows that the differences in health literacy, social support, and health empowerment scores among different categories of older people comorbidities are statistically significant (*p* < 0.05). The logistic regression result showed that health literacy, social support, and health empowerment scores are the risk factors for self-management scores among older comorbidity. The results are consistent with the results of related studies in China [12, 29, 30]. Patients with high health literacy have good information acquisition ability, they are able to find what they need from a large amount of information, grasp the main conflicts, and have better medical compliance behaviour.So their self-management level is higher [30]. Patients with a high level of social support receive a high level of spiritual and material support from the social support system, it is conducive to promoting changes in their health behaviors and improving their self-management level [31]. Older people health empowerment is a positive self-care strategy and cooperative relationship. Elderly patients with chronic diseases gradually manage themselves and empower others by stimulating internal responsibility and obtaining external social support, so as to achieve the goal of improving health outcomes and quality of life [32]. Empowerment can help patients discover and develop their inner potential for chronic disease management, enabling them to better assume full responsibility for self-management. It in turn promotes behavioral change [33]. In summary, it is recommended that healthcare professionals enrich the form of health education and add some fun activities that are educational and entertaining. It can increase the experience of the older people, better enhance the enthusiasm and educational effect of the older people in participating in health education [34].This can increase the experience of the older people and improve their motivation to participate in health education and the effectiveness of education, it in turn improves their health literacy and health empowerment. It is conducive to the improvement of their self-management level. At the same time, we can consider adopting different forms of health education for different groups of people.Such as adopting information technology means of health education for the older people who are more receptive to electronic devices, adopting traditional face-to-face forms for the older people who are older and more conservative in their thinking, so as to simplify the form and content of education. In addition, in terms of social support, help the older people to build a perfect social support network, encourage the older people to actively participate in group activities, and provide emotional and material support for the older people from the perspective of family members, peers, and patients, so as to enhance the older people's confidence in disease management. It is conducive to the catharsis of the older people's stress and the improvement of health behaviors, and to enhance their self-management ability.

The results of this study shows that the difference in e-health literacy is statistically significant among older co-morbid patients with low self-management type and medium self-management type (*p* < 0.05). The possible reason for this is that the higher e-health literacy level of older people people's ability to acquire, understand, and critique online health knowledge. It is more conduciv to accumulate healthy lifestyles and adopt correct health behaviors [35]. Studies have shown that higher e-health literacy levels help individuals to perform disease self-management, improve health behaviors, and enhance quality of life [36]. However, the result of this study shows that the overall level of e-health literacy among older comorbidity patients is not optimistic, and it has become an important factor that restricts the improvement of self-management in the older people. It is consistent with the results of related studies [37]. Therefore, community healthcare workers should train the use of electronic devices for different older people characteristics and popularize internet knowledge so that the older people can enjoy the benefits of internet devices. It is conducive to enhance their acceptance of smart devices, improve the e-health literacy level of the older population, and then enhance their self-management level.

## Limitation

This study still has some limitations:It is a cross-sectional survey, the sample is only from Zhengzhou City, Henan Province, which has some geographical limitations and limited generalization, and a multi-centre, large-sample survey can be carried out in the future, so that the results of the study can be more generalized. Meanwhile,longitudinal research can be considered to explore the changes of self-management ability of the elder over time. In addition,other factors influencing the self-management ability of elderly with comorbidity, beyond those assessed in this study; for example, types and duration of comorbidity can significantly influence self-management ability of older co-morbid patients.These factors should be incorporated in future studies examining self-management ability of older co-morbid patients. Despite these limitations, this study was able to show the benefits of using LPA to identify self-management profiles and examined Influencing factors of self-management ability of different types of older co-morbid patients. This study provides new insights for the research field of comorbidity in the elderly and provides reference for personalized intervention in the later period.

## Conclusion

The self-management characteristics of older co-morbid patients were classified into three categories by LPA: low self-management, medium self-management, and good self-management. There are differences in health literacy, health empowerment, and social support levels among different categories of older co-morbid patients. Our findings may help community workers to pay attention to the elderly patients with low self-management ability and provide them with targeted and actionable intervention measures to improve their self-management ability and improve their quality of life.

## Data Availability

The datasets generated and/or analysed during the current study are not publicly available due to the data containing information that could compromise research participant privacy/consent, but are available from the researcher Wu Lanxin (1094667097@qq.com) on reasonable request, and subject to approval from the research committee of the Zhengzhou University.
